# The nexus between health status and health expenditure, energy consumption and environmental pollution: empirical evidence from SAARC-BIMSTEC regions

**DOI:** 10.1186/s12889-021-11534-w

**Published:** 2021-09-16

**Authors:** Mohammad Mafizur Rahman, Khosrul Alam

**Affiliations:** 1 School of Business, University of Southern Queensland, Toowoomba, Australia; 2 Department of Economics, Bangabandhu Sheikh Mujibur Rahman Science and Technology University, Gopalganj, 8100 Bangladesh

**Keywords:** Health status, Energy consumption, Environmental pollution, Health expenditure, Panel ARDL, I10, I15, I18, C23

## Abstract

**Background:**

The COVID-19 pandemic has highlighted the need for the betterment of health status, while also considering health expenditure, energy, and environmental issues. This paper examines the nexus between health status and health expenditure (both public and private), energy consumption and environmental pollution in the SAARC-BIMSTEC region.

**Methods:**

We utilized the panel autoregressive distributed lag (ARDL) model, the heterogeneous panel causality test, the cross sectional dependence test, the cointegration test and the Pesaran cross sectional dependent (CADF) unit root test for obtaining estimated results from data over 16 years (2002–2017).

**Results:**

Our results authorize the cointegration among the variables used, where the coefficients of energy consumption, public and private health expenditures, and economic growth are 0.027, 0.014, 0.030, and 0.029, respectively, and indicating positive and statistically significant effects. The coefficient of environmental pollution is − 0.085, implying significant negative effect on the health status of these regions in the long-run. However, no panel wise significant impact is found in the short-run. Bidirectional and unidirectional causal links between the studied variables and the health status are also identified..

**Conclusions:**

The improved health status in the SAARC-BIMSTEC region needs to be protected by articulating the effective policies. The attained results are theoretically and empirically consistent, and have important policy implications in the health sector.

## Introduction

In 2015, the United Nations (UN) proposed Sustainable Development Goals (SDGs) to be achieved by 2030, citing good health and well-being as one of the principal targets, and incorporating affordable and clean energy, pure water and sanitation to improve the health of people, no matter where they live (Goal-3, 6 and 7, [1, 2]). Good health is a fundamental human right; however, recent and previous pandemics and epidemics have highlighted the need to re-think a number of health-related issues globally. The world is now experiencing a huge toll on health and life due to the ignorance and lack of proper consideration of various health related factors like energy, environment, public and private health expenditures, hygiene, and sanitation. In many areas, health facilities have been found to be wanting, exacerbating those health-related issues. Therefore, ensuring better health facilities for all people to build a safer world has now become a prime policy goal across the globe. The challenges resulting from COVID-19 have made the implementation of these goals imperative.

In the SAARC-BIMSTEC region, health status, health expenditures, energy use, the environment and economic growth are essential considerations that require prioritization. This region comprises of ten emerging countries^1^ covering a combined geographical area of 5,935,573.999 km^2^ with a total population of 1959.45 million, many of whom are living in poverty (20.54% approximately), where awareness of health issues is low and therefore requires serious attention [3]. In 2017, the average life expectancy at birth in this region was 71.18 years, the highest belonging to Thailand (76.68 years) and the lowest belonging to Afghanistan (64.13 years) [3]. The average public and private health expenditures in 2017 were 38.27 and 55.15% respectively, out of the total current health expenditures (5.05% of GDP) in this region, which is an insignificant amount when placed into context [3]. The average economic growth of this region in early 2017 was 5.63%, which improved over the year. Increased industrialization and urbanization may have played a critical role in this improvement [3]. In terms of energy utilization and carbon discharges in this region, the average per capita energy intake and CO_2_ emissions in 2017 were 697.61 kg of oil equivalent and 1.36 metric tons [3–5]. As economic growth is increasing,, environmental pollution is also on the rise in these regions, which may exacerbate health condition of people. Therefore, careful attention on the issues of economic growth, public and private health expenditure, energy consumption and environmental pollution is crucial for the betterment of public health.

Previous studies that considered health status were largely inconclusive and thus, were of no help in formulating cohesive policies in the health sector. The differences of opinion may be due to the variety of methodologies, regions, countries, variables, and data, which create an enigmatic nexus between the related variables and the health status, creating the opportunity for further investigations for formulating and executing proper policy strategies. In this regard, our study is an effort to identify the decisive impact of health expenditure (both public and private), energy consumption and environmental pollution on the health status by utilizing various voguish econometric tools in the case of the panel of SAARC-BIMSTEC countries.

The key objectives of this study are:

i. To identify the impact of health expenditure (public and private), CO_2_ emissions, energy consumption, sanitation facilities, and economic growth on the health status in the SAARC-BIMSTEC region.
ii. To detect the long-term and short-term associations, causality and dynamics among health status, health expenditure (public and private), CO_2_ emissions, energy consumption, sanitation facilities, and economic growth.

The major contributions of this study are: (i) this is the first study, to the best of our understanding, that investigates the effects of public and private health expenditures, energy consumption, CO_2_ emissions, economic growth, and sanitation facilities on the health status in the context of SAARC-BIMSTEC regions; (ii) the results are attained by employing various erudite econometric tools e.g. cross-sectional dependence test, CADF panel unit root test, Pedroni and Kao test, Pool Mean Group (PMG) or panel autoregressive distributed lag (ARDL) method, Dumitrescu and Hurlin causality test; and (iii) the outcomes will provide guiding principles for policy makers to ensure improved health status by espousing massive health expenditures (public and private), lower carbon emissions and energy consumption, and effective economic growth policies.

The study is designed in the following order: following the introduction, section 2 displays methods; section 3 explicates the results; section 4 analyzes the discussions; and section 5 presents the conclusion and policy implications.

## Methods

### Literature review

Previous studies have made an effort to assess and derive the most important factors relating to health and its nexus with the other elements that have been previously mentioned. Factors affecting health status are various. In this study, we will review the past literature in the context of our research objectives.

Various researchers ([6–10], among others) have noted the increased health expenditure that benefits health indicators like life expectancy, infant and child mortality rate, and crude death rate [6]. pointed out the significant positive effect of health care expenditure on life expectancy in developed countries due to well-judged spending; they however found the insignificant impact for developing countries because of lack of focus on quality rather than quantity of expenditure.). Similarly, applying panel data estimation technique, [7] identified the positive impact of health expenditure on life expectancy in 175 countries during 1995–2010.. They also obtained significant country effects and important differences among the countries. In the same way, [8] ascertained the positive effect of health care expenditure on life expectancy at birth for both males and females, where the fixed effect model was used for 210 countries and regions over the period from 1995 to 2014 [9]. provided evidence that health expenditure positively affects health outcomes in the presence of high institutional quality in the context of 18 Middle Eastern and North African (MENA) countries for the period from 1995 to 2012, although they considered health expenditure to be a necessary but not sufficient requirement for better health status [10]. also identified the significant influence of health expenditure on reducing chronic diseases, which results in lengthening life expectancy in the data of 13 countries with the instrumentation of parliament political composition.

In contrast, a number of researchers found unclear or insignificant relationships between health expenditure and health indicators (see [11–13], among others). In this context, [11] conducted a study on the health care expenditure and the health outcomes nexus in the SAARC-ASEAN regions for the data period of 1995–2014. They employed fixed effects, random effects and generalized method of moments (GMM) approaches, where they identified the positive impact of health expenditure on crude death rate and infant mortality rate but no significant impact on life expectancy at birth [13]. found that health care expenditure did not improve health and advocated reallocating public health expenditure from medical expenditures to social programs in the USA to raise life expectancy and reduce death [12]. found a varying effect of health care spending on life expectancy due to the heterogeneity of social development across the 31 provinces of the China over the period 2000–2010 by employing geographically weighted regression analysis. Similarly, [14] did not clearly observe any nexus between health expenditures and the length of life in case of the OECD from a sample of 560 pooled time-series and cross-section observations [15]. identified per capita health expenditure as a weak determinant whereas literacy, per capita income, and access to safe water supplies were identified as strong positive determinants on life expectancy from the multivariate cross-national analysis of 1990.

Ongoing debates show that there are conflicting opinions on whether emphasis should be on public or private health expenditure for ensuring better health facilities. In this context, [16] found a positive effect of public health expenditure but a negative effect of private health expenditure on the life expectancy at birth for 195 countries for the data period of 1995–2014 where they used generalized method of moments (GMM) estimation technique. Using panel regression model for the data of 2000–2015, [17] also revealed that public health expenditure had a significant impact but private health expenditure had little or no significant impact on life expectancy in the context of three groups of selected middle or high income countries.. Similarly, [18] found a higher impact of public health expenditure but a lower impact on life expectancy in the OECD countries over the period 1980–2000, while using cross-country fixed effects multiple regression analysis [19]. revealed that higher public health expenditure lowered the mortality rate in Italy over the period 1999–2013; they applied a pooled cross-sectional time series study. On the other hand, employing ordinary least squares (OLS) regression, [20] identified the positive and statistically significant influence of private health expenditure but no significant impact of public health expenditure on life expectancy in Cameroon during 1980–2014; they also obtained the bidirectional and unidirectional causal links of private and public health expenditure, respectively. Similarly, while conducting a study in 31 European countries for the data period of 2014–2014, [21] found that although public health expenditure is not a key determinant of life expectancy, the social protection expenditures are important factors. A simple linear regression model was used for the study. Many other researchers pointed out the positive role of both public and private health care expenditures on health status. For example, [22] presented the strong positive link of both public and private health care expenditure with life expectancy, although public health spending had comparatively greater influence in case of 44 countries in sub-Saharan Africa using the panel data of 1995–2010 from the fixed and random effects panel regression models [23]. also found that both the public and private health expenditures reduced the maternal mortality rate, the under-five mortality rate, and the infant mortality rate in the MENA region because of effective government and private health spending on health care during 1990–2010. These findings were achieved with the aid of pooled ordinary least regression, random effects, and Hausman-Taylor instrumental variable models. Thus, the inconclusive interconnection between health expenditure and health status demands further investigation.

Energy is the life blood of the modern world, ensuring better living for people by making technological amenities available, and providing sophisticated and improved medical facilities. Energy consumption is another important factor that warrants investigation. Recently, several researchers ([24–27], among others) have tried to ascertain the link between health status and energy consumption [24]. discovered that energy consumption reduced and carbon emission increased child mortality in 23 African countries over the period 1999–2014.The findings were based on the baseline pooled OLS and system-generalized method of moments (GMM) estimators. Examining this concept in more detail, [25] found that non-renewable energy consumption adversely affected life expectancy by raising the mortality rate, while renewable energy consumption reduced the mortality rate and extended life expectancy in 34 Sub-Saharan African countries where system- generalized method of moment (GMM) analysis was used over the period of 1995–2015. In the same way, using the bootstrap panel analysis of causality, [26] found a strong link between health and energy consumption in Africa, where in some African countries they found unilateral causality between energy use and life expectancy, and under-5 child mortality from the data of 1971–2010. Furthermore, [27] employed longitudinal analysis techniques and found that declining energy intensity and increasing energy efficiency improved human well-being in the 12 Central and Eastern European (CEE) countries for the period of 1992–2010. Thus, further investigation on the link between energy consumption and health status is required for correct policy initiatives.

The effect of environmental pollution on health is now becoming another key concern, and some research relates clearly to this vital issue (see [8, 28–35],among others). Applying the panel least squares method, [28] found the negative impact of CO_2_ emissions on life expectancy at birth in 25 EU countries covering the period 1995–2013. Similarly, [29] conducted a study on the effect of income inequality, globalization and environmental degradation proxied by CO_2_ in Pakistan, covering the period 1980–2015. Using the auto regressive distributive lag (ARDL) model, they ascertained the significantly negative impact of environmental degradation on life expectancy [30]. investigated the link between health financing, CO_2_ emissions and life expectancy in Nigeria. Applying Bayer and Hanck’s cointegration test for the data period 1970–2014, they found cointegration among variables but identified an insignificant negative impact of CO_2_ emissions on life expectancy at birth. Similarly, by applying fixed effects and random effects models, [8] discovered an insignificant effect of CO_2_ emissions on average life expectancy at birth in 210 countries and territories [33]. conducted a study on the impact of environmental factors on health status in 12 countries of Southern African Development Community (SADC) during the period 2000–2008, where they considered CO_2_ emissions per capita, access to improved water source, and access to improved sanitary facilities to measure environmental quality, and annual infant mortality rate to measure health status. By employing fixed effects and random effects regression analysis, they found that two environmentally related variables: access to water and sanitary facilities had significant effects on reducing infant mortality whereas CO_2_ emissions had an insignificant effect. Similarly, considering mortality as an indicator of public health status, [34] identified that CO_2_ emissions significantly increase the death rate in case of top 20 industrialised countries of the world.

On the other hand, [36] obtained a significantly positive impact of CO_2_ emissions on life expectancy in West Africa for the data period of 2000–2018 by applying two stages least squares econometric techniques [32]. found an insignificant positive impact of CO_2_ emissions on life expectancy in 136 countries covering the period 2002–2010. Similarly, [31] employed ordinary least square (OLS) techniques and also found an insignificant positive linkage between CO_2_ emissions and life expectancy in Nigeria over the period of 1995–2013. In addition, [35] found a bidirectional causal association between CO_2_ emissions and infant mortality rate in India which validated the feedback causality. Furthermore, [37] found that the life expectancy increases while death rate reduces economic growth under the COVID-19 pandemic situation in Nigeria over the data period of 1989–2018.. Similarly, [38] found the reduction in CO_2_ emissions while conducting a study considering the consequences of COVID-19 on the social isolation in the Chinese economy [39]. also noted that under global pandemic (COVID-19) condition, the necessary arrangements of tele-health facilities can play significant role in improving the human health in case of African nations. However, they did not focus on various factors like public and private health expenditures, CO2 emissions and energy consumption explicitly on human health status. Therefore, the study relating to the interconnection between environment and health status is appealing and deserves more thorough investigation.

From the exploration of the aforementioned literature, it has been observed that the results are unconvincing and not encouraging to the expression of any appropriate policy initiatives towards ensuring better health status for the people. Moreover, the combined consideration of both public and private health expenditures, energy consumption and environmental pollution on health status is absent in most of the literature, especially in the SAARC-BIMSTEC region. Therefore, this study will fill the existing gaps and provide an expedient way of probing the impact of health expenditure, energy consumption and environmental pollution on health status and the articulation of proper policy implications.

### Study selection

#### Theoretical or empirical rationale for choosing the variables

The theoretical justification for this study may be linked with various well-known theories e.g. human capital theory suggested by [40] ([24, 41, 42]); the health care model recommended by [43, 44] ([22, 24, 40, 43, 44]); and the inclusion of human capital in the neoclassical endogenous growth model by [45] ([22, 45]).

The core rationale for selecting the variables is:: (i) a combination of public and private health expenditures ensure better health status; (ii) energy consumption creates enough facilities to obtain good health status; (iii) excessive carbon emissions are detrimental to better health outcomes; (iv) economic growth offers improved amenities for safeguarding health status; and (v) effective sanitation facilities contribute to improved health status.

The empirical rationale for selecting our variables is mainly on the basis of the data availability, and past and existing literature. We have accessed the data on the variable life expectancy at birth to determine the health status following [11, 16, 20, 46], and others; public and private health expenditures are considered following [7, 8, 11], and others; energy consumption in the line of [24–27], among others; the carbon emissions to denote environmental pollution in the line of [8, 30, 31, 35], among others; per capita GDP as a proxy of economic growth following [11, 24], and others, and the sanitation facilities following [11, 22], among others.

The identification of our variable is as follows:

- HS: Health status
- PUH: Public health expenditure
- PVH: Private health expenditure
- ENC: Energy consumption
- CO_2_: Carbon emissions
- PerGDP: Per capita gross domestic product
- SAN: Sanitization facilities

#### Variables and data

To conduct our study, we have utilized the health status as a dependent variable; as a proxy of this, variable life expectancy at birth is used. The independent variables are public and private health expenditures, carbon emissions, energy consumption, sanitation facilities, and economic growth. Both public and private health expenses are taken as a percentage of current health expenditures; energy consumption is considered to be a per capita oil equivalent, and the carbon emissions variable is taken as metric tons per capita. Per capita gross domestic product (GDP) is used as the proxy variable for economic growth, the, and the sanitation variable is considered as the basic sanitation use by the % of total population.

In this study, we used data provided by the World Development Indicator [3] from the World Bank for the years 2002–2017. However, due to the lack of data of energy use in case of Afghanistan, Bhutan and the Maldives, we have taken figures from the data (2002–2017) of [5] data world atlas. For all other countries beyond WDI, carbon emission and energy consumption data for the period of 2015–2017 were collected from [5] data world atlas and [4] statistical review. For our estimation, we have used two well-known statistical software packages, STATA-16 and E-views-10.

The model used for empirical estimation is depicted below:

1 $$ \mathrm{HS}=\mathrm{f}\ \left(\mathrm{PUH},\mathrm{PVH},\mathrm{ENC},{\mathrm{CO}}_2,\mathrm{PerGDP},\mathrm{SAN}\right) $$

After converting all the variables of the eq. (1) into natural logarithmic form, we will obtain the elasticity of every variable from the coefficient values. Therefore, the eq. (1) can be depicted as:

2 $$ {\mathrm{LNHS}}_{\mathrm{t}}=\upalpha +{\upbeta}_1{\mathrm{LNPUH}}_{\mathrm{t}}+{\upbeta}_2{\mathrm{LNPVH}}_{\mathrm{t}}+{\upbeta}_3{\mathrm{LNENC}}_{\mathrm{t}}+{\upbeta}_4\mathrm{LNCO}{2}_{\mathrm{t}}+{\upbeta}_5{\mathrm{LNPerGDP}}_{\mathrm{t}}+{\upbeta}_6{\mathrm{LNSAN}}_{\mathrm{t}}++{\upvarepsilon}_{\mathrm{t}} $$

Where, α is the intercept, and β_1_, β_2_, β_3_, β_4_, β_5_, β_6_ are coefficients and ε_t_ is the error term.

#### Econometric approach

In this study we applied various econometric techniques for the purpose of estimation. We employed the following tests: a cross-sectional dependence test to observe the shock effect; CADF panel unit root test to confirm the stationarity of variables; the [47–49] cointegration tests to measure the equilibrium association; the panel pooled mean group (PMG) or panel ARDL method to estimate the long and short term equilibrium effects; and [50] heterogeneous panel causality to discover the direction of causality.

##### Cross-sectional dependence

Cross-sectional dependence of the variables may appear due to the prevalence of analogous geographic, economic, historical, ethnic and political jolts; hence these jolts should be checked before the detection of the unit root. In this study, we have utilized four renowned cross-sectional dependency tests: [51] BP LM, [52] scaled LM, [52] CD, and [53] biased-corrected scaled LM.

To examine the cross-sectional dependence among the panel data, the below model is suggested by [51]:

$$ {CD}_{BP}=\sum \limits_{i=1}^{N-1}\sum \limits_{j=i+1}^N{\hat{p_{ij}}}^2 $$

To overcome the limitations of the above, [52] develops the following LM statistics:

$$ {CD}_{LM}=\sqrt{\frac{1}{N\left(N-1\right)}}\ \sum \limits_{i=1}^{N-1}\sum \limits_{j=i+1}^N\left({\hat{p_{ij}}}^2-1\right) $$

[52] further recommends that if the cross-sectional size is greater than the time dimension, the following test statistic can be used instead:

$$ CD=\sqrt{\frac{2T}{N\left(N-1\right)}}\ \sum \limits_{i=1}^{N-1}\sum \limits_{j=i+1}^N{\hat{p_{ij}}}^2 $$

From the simple asymptotic bias correction, the scaled LM test as recommended by [53] is:

$$ {CD}_{BC}=\sqrt{\frac{1}{N\left(N-1\right)}}\ \sum \limits_{i=1}^{N-1}\sum \limits_{j=i+1}^N\left({\hat{p_{ij}}}^2-1\right)-\frac{N}{2\left(T-1\right)} $$

Where $\hat{p_{ij}}$ specifies a correlation among the errors. In this test the null hypothesis is H_0_: no cross-sectional dependence whereas the alternative hypothesis is H_1_: cross-sectional dependence.

##### Panel unit root test

Detection of unit root in the series of variables is essential to estimate it in the PMG or panel ARDL model. In this regard, we have used the cross-sectional augmented Dickey-Fuller (CADF) test because of the consideration of cross-sectional panel data following the methodology of [54] under the following equation:

$$ \Delta  {x}_{it}={\alpha}_i+{p}_1{x}_{it-1}+{\\delta}_1{\overline{x}}_{t-1}+\sum \limits_{j=0}^n{\eta}_{ij}\Delta  {\overline{x}}_{it-1}+\sum \limits_{j=0}^k{\Psi}_{ij}\Delta  {x}_{it-1}+{\varepsilon}_{it} $$

Where, ${\overline{x}}_{t-1}$ and $\Delta  {\overline{x}}_{it-1}$ indicate the cross-sectional averages of lagged levels and first difference individual series respectively.

##### Co-integration test

For co-integration among the variables, a residual based cointegration test as advised by [47, 48] has been employed on the basis of two varieties: panel test and group test. The panel test has four statistics under the within dimension: panel PP, panel rho, panel v and panel ADF, whereas the group test has three statistics under the between dimension: group PP, group rho and group ADF. It is considered that the mentioned statistics are standard, normally and asymptotically distributed residuals based on the following long-term model:

$$ {Y}_{it}={a}_i+{\zeta}_i+\sum \limits_{j=1}^m{\varPsi}_{ji}{X}_{ji t}+{\varepsilon}_{it} $$

The construction of the appraised residuals is as under:

$$ {\varepsilon}_{it}={p}_i{\varepsilon}_{it-1}+{u}_{it} $$

The comparison is made based on the maximum likelihood panel cointegration statistics considering no co-integration among the variables as the null hypothesis. The cointegration system for panel data recommended by [47, 48] is as follows:

$$ {Y}_{it}={a}_i+\varPsi {X}_{it}+{\varepsilon}_{it} $$

We have also used another co-integration test to observe the homogenous nexus as suggested by [49] under two types of test: the Dickey-Fuller and the Augmented Dicky Fuller, where no co-integration among the variables as the null hypothesis is assumed.

##### Pool mean group or panel ARDL estimation

To identify the dynamic nexus between health status and public and private health expenditures, carbon emissions, energy consumption, sanitation facilities, and economic growth, we have utilized the pool mean group **(**PMG) or panel autoregressive distributed lag (ARDL) method under the following equation:


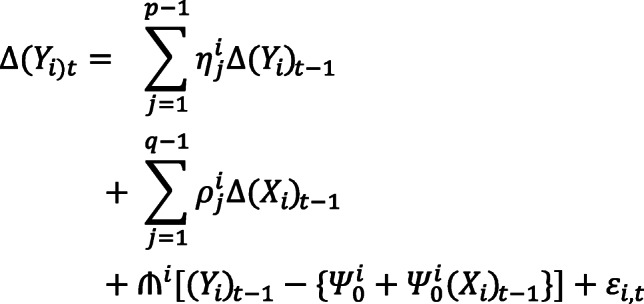


Where, Y is the health status. X is the set of explanatory variables comprising public and private health expenditures, energy use, CO_2_ emissions, economic growth, and sanitation facilities. The short-term coefficients of dependent and independent variables are symbolized respectively by *η* and *ρ*, where the long-term coefficients are denoted by *Ψ*. The speed of adjustment towards long-term affiliation is denoted by *₼*, and the time-varying error term by *ε* .  We have used the subscript i to determine country and t for time. The long-term growth regressions are shown within the square brackets.

##### Heterogeneous panel causality test

To perceive the short-term bivariate causal association among the variables we have adopted the [50] heterogeneous panel causality test that considers the cross-sectional dissimilarities under the following model:

$$ {y}_{it}={a}_i+\sum \limits_{j=1}^J{\eta_i}^J{y}_{i,t-J}+\sum \limits_{j=1}^J{\rho_i^J}_i{x}_{i,t-J}+{\varepsilon}_{i,t} $$

The x_i,t_ and y_i,t_ denote the observations of two stationary variables of individual i in period t, j exposes the lag length, *η*_*i*_^(*J*)^ displays the autoregressive parameter, and ${\rho}_i^{(J)}$ designates the regression coefficient varying within the groups. The identical lag order J for all individuals is considered so that the panel may be balanced. It is a fixed coefficient, normally distributed and permits the case of heterogeneity.

This test assumes no causality as the null hypothesis and the existence of causality between variables as the alternative hypothesis, as under: 
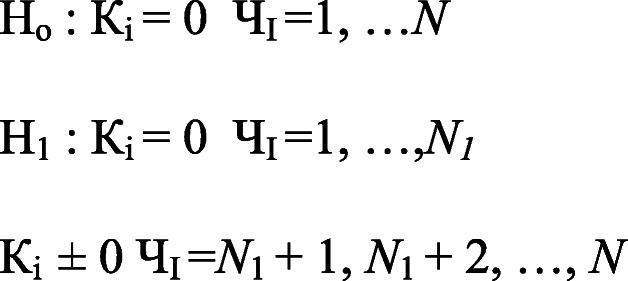


Here, *N*_1_ represents the unknown parameter that fulfils the condition 0 ≤ *N*_1_ /*N* < 1. The ratio of *N*_*1*_/*N* ought to be lower than 1, because N_1_ = 0 indicates causality for all individuals in the panel, whereas, N_i_ = N means no causality, so that we failed to reject the null hypothesis.

## Results

### Descriptive statistics

In Table 1, the results of the descriptive statistics of our considered variables are displayed in natural logarithmic form. We have found that the mean, median, maximum, minimum, and standard deviation values of the health status are 4.224, 4.218, 4.361, 4.039, and 0.076 respectively. The mean values of public health expenditure, private health expenditure, energy consumption, CO_2_ emissions, per capita GDP, and sanitation facilities are, consecutively, 3.263, 3.966, 6.080, − 0.398, 7.289, and 4.016, and their standard deviations are, consecutively, 0.827, 0.494, 1.032, 1.093, 0.893, and 0.431.

**Table 1:** Descriptive statistics

	LNHS	LNPUH	LNPVH	LNENC	LNCO_2_	LNPERGDP	LNSAN
Mean	4.224	3.263	3.966	6.080	−0.398	7.289	4.016
Median	4.218	3.231	4.221	6.120	−0.406	7.060	4.055
Maximum	4.361	4.336	4.455	7.700	1.530	8.963	4.599
Minimum	4.039	−0.115	2.661	2.979	−3.259	5.800	2.989
Std. Dev.	0.076	0.827	0.494	1.032	1.093	0.893	0.431
Observations	160	160	160	160	160	160	160

Note: All the variables are converted into the natural logarithm form

### Cross-sectional dependence test results

We have reported the values of four types of cross-sectional dependence tests and their probabilities in Table 2. All the variables except public health expenditure have confirmed the significance in all four tests, which has assured three tests. From the Table 2 we can observe that the probability values of our studied variables are at different levels and can therefore reject the null hypothesis of cross-sectional independence.

**Table 2:** Cross-sectional dependence test results

Variables	Breusch-Pagan LM	Pesaran scaled LM	Bias-corrected scaled LM	Pesaran CD
LNHS	705.850*** (0.000)	69.600*** (0.000)	69.326*** (0.000)	26.566*** (0.000)
LNPUH	171.068*** (0.000)	13.289*** (0.000)	12.955*** (0.000)	−0.888 (0.374)
LNPVH	213.210*** (0.000)	17.731*** (0.000)	17.798*** (0.000)	−1.942* (0.052)
LNENC	261.927*** (0.000)	22.866*** (0.000)	22.533*** (0.000)	9.781*** (0.000)
LNCO_2_	337.622*** (0.000)	30.845*** (0.000)	30.512*** (0.000)	16.745*** (0.000)
LNPERGDP	640.482*** (0.000)	62.769*** (0.000)	62.436*** (0.000)	25.257*** (0.000)
LNSAN	714.776*** (0.000)	70.601*** (0.000)	70.267*** (0.000)	16.056*** (0.000)

Note: ***, **, and * denote significance level at 1, 5 and 10%, respectively. Figures in the parentheses are probabilities

### Unit root test results

To detect the presence of the unit root we have employed the cross-sectional ADF unit root test (CADF) as per the methodology of [54], due to the contemplation of the cross-sectional dependence issue. By applying the constant only we have found that the LNHS, LNPUH, and LNSAN have no unit root at level, where the LNENC, LNCO_2_, LNPVH, LNPERGDP have unit root at level but no unit root at their first difference (Table 3). As per the methodology of [55], we may utilize the ARDL model in case the series has no unit root or stationary at level I(0), at first difference I(1) or both, but none may be stationary at secondary difference I(2).

**Table 3:** Unit root test results

Variables	Constant				Order of Integration
	Level	*p*-value	1st difference	p-value	
LNHS	−5.248***	0.000	−2.229*	0.066	I(0)
LNENC	−1.840	0.368	−3.272***	0.000	I(1)
LNCO_2_	−1.578	0.680	−3.381***	0.000	I(1)
LNPUH	−2.399**	0.020	−3.772***	0.000	I(0)
LNPVH	−2.047	0.165	−3.313***	0.000	I(1)
LNPERGDP	−1.374	0.863	−2.955***	0.000	I(1)
LNSAN	−3.189***	0.000	−2.335**	0.035	I(0)

Note: ^***, **,^ and ^*^ denote significance level at 1, 5 and 10%, respectively

### Cointegration test results

To investigate the prevalence of the long-term nexus between health status and health expenditure (both public and private), energy consumption, environmental pollution, economic growth, and sanitation we have taken the assistance of two kinds of panel cointegration tests as proposed by [47–49]. Both tests have assured the existence of long-term cointegration among our studied variables, and are therefore suitable to evaluate the parameters of the dynamic error correction model by applying the panel ARDL model or the PMG method (Table 4).

**Table 4:** Pedroni and Kao panel cointegration test results

Pedroni cointegration test				
Estimates	Statistic	Prob.	Weighted Statistics	Prob.
*Alternative hypothesis: common AR coefficients (within-dimension)*				
Panel v-Statistic	−0.837	0.799	0.347	0.3642
Panel rho-Statistic	3.456	0.999	2.644	0.996
Panel PP-Statistic	0.522	0.699	−1.614*	0.053
Panel ADF-Statistic	−1.821**	0.034	−2.554***	0.005
*Alternative hypothesis: individual AR coefficients (between-dimension)*				
Group rho-Statistic	3.843		0.999	
Group PP-Statistic	−3.082***		0.001	
Group ADF-Statistic	−2.995***		0.001	
Kao cointegration test				
Statistic	t-statistic		Prob.	
ADF	−3.593***		0.000	

Note: ^***, **,^ and ^*^ denote significance level at 1, 5 and 10%, respectively

### The results of panel ARDL estimation

From the model of PMG or panel ARDL(1,1,1,1,1,1,1) and applying Akaike information criterion in the case of constant (level) case-II, we found the long-term and short-term association among our studied variables (Table 5). The optimal lag orders of ARDL (1,1,1,1,1,1,1) model is selected based on Akaike information criterion. We observed that the coefficients of the public and private health expenditures and energy consumption are, respectively, 0.014, 0.030, and 0.027, which are positive and statistically significant at 1% level. The coefficient of environmental pollution proxied by CO_2_ is − 0.085, which is negative and statistically significant at 1% level. The coefficients of economic growth and sanitation facilities are 0.029 and 0.018, where the former is statistically significant at 5% level but the latter is insignificant. The coefficient of error correction term (COINTEQ01) is − 0.034, which is negative and statistically significant even at 1% level assuring long-term cointegration among the considered variables. However, in the short-term we found that neither variable is statistically significant, which denotes that the fruit of the above variables on health status cannot be found instantaneously and will have to wait for the long term.

**Table 5:** PMG or panel ARDL results

Variable	Coefficient	Std. Error	T-Statistic	Prob.
Long Term				
LNPUH	0.014***	0.005	2.691	0.009
LNPVH	0.030***	0.011	2.821	0.006
LNENC	0.027***	0.009	3.095	0.003
LNCO_2_	−0.085***	0.024	−3.568	0.001
LNPERGDP	0.029**	0.012	2.368	0.021
LNSAN	0.018	0.069	0.261	0.795
Short Term				
COINTEQ01	−0.034***	0.012	−2.848	0.0057
D(LNPUH)	−0.001	0.001	−0.702	0.485
D(LNPVH)	−0.005	0.004	−1.240	0.219
D(LNENC)	0.0004	0.0004	1.021	0.311
D(LNCO_2_)	0.003	0.002	1.616	0.110
D(LNPERGDP	−0.007	0.004	−1.604	0.113
D(LNSAN)	−1.122	1.019	−1.101	0.274
Constant	0.124**	0.048	2.587	0.0117

Note: ^***, **,^ and ^*^ denote significance level at 1, 5 and 10%, respectively

### Country specific short-term test results

The outcomes of the country specific tests in the short-term are displayed in Table 6. The findings are mixed which are analysed in the discussion section.

**Table 6:** Short-term country specific results of all sample countries

Country	COINTEQ01	LNPUH	LNPVH	LNENC	LNCO_2_	LNPERGDP	LNSAN	Constant
Afghanistan	0.012***	−0.001***	0.001***	0.00***	0.001***	0.001***	0.523***	−0.055***
Bangladesh	−0.03***	−0.003***	− 0.016***	0.001***	0.006***	0.018***	0.035**	0.098***
Bhutan	0.0121***	0.005***	−0.0002***	0.003***	−0.0001***	−0.002***	0.877***	−0.052***
India	−0.080***	0.003***	0.009***	−0.001***	0.002***	−0.010***	−0.089***	0.302***
Maldives	−0.058***	0.001***	−0.001***	−0.001***	0.002***	−0.001***	0.0217***	0.218***
Nepal	−0.006***	−0.0002***	− 0.001***	−0.000***	0.0002***	−0.001***	0.057***	0.024***
Pakistan	−0.066***	0.000***	−0.002***	0.000***	−0.002***	−0.014***	− 0.098***	0.241***
Sri Lanka	−0.090***	−0.004***	− 0.032***	0.002***	0.017***	−0.036***	−6.245	0.378***
Myanmar	−0.020***	0.0003***	0.002***	−0.0004***	0.001***	−0.018***	−7.951*	0.032***
Thailand	−0.014***	−0.010***	− 0.006***	−0.001***	0.002***	−0.008***	1.689**	0.051***

Note: ^***, **,^ and ^*^ denote significance level at 1, 5 and 10%, respectively

### Results of the Dumitrescu and Hurlin panel causality test

In Table 7, the [50] panel causality outcomes are shown by w-statistic and corresponding probability. In this study, we have observed the bidirectional causality between health status, and health expenditures (both public and private), carbon emissions, economic growth, and sanitation facilities, but unidirectional causality emanating from the energy use to health status.

**Table 7:** Causality test results

Null Hypothesis:	W-Stat.	Prob.	Decision
LNPUH does not cause LNHS	3.495***	0.000	LNPUH ↔ LNHS (bidirectional causality)
LNHS does not cause LNPUH	2.783**	0.012	
LNPVH does not cause LNHS	3.786***	0.000	LNPVH ↔ LNHS (bidirectional causality
LNHS does not cause LNPVH	2.454**	0.046	
LNENC does not cause LNHS	5.315***	0.000	LNENC→ LNHS (unidirectional causality)
LNHS does not cause LNENC	1.623	0.501	
LNCO_2_ does not cause LNHS	9.660***	0.000	LNCO2↔ LNHS (bidirectional causality)
LNHS does not cause LNCO_2_	3.265***	0.000	
LNPERGDP does not cause LNHS	9.837***	0.000	LNPERGDP ↔LNHS (bidirectional causality)
LNHS does not cause LNPERGDP	3.094***	0.003	
LNSAN does not cause LNHS	19.951***	0.000	LNSAN ↔ LNHS (bidirectional causality)
LNHS does not cause LNSAN	37.283***	0.000	

Note: ^***, **,^ and ^*^ denote significance level at 1, 5 and 10%, respectively

## Discussion

Our main findings (see Table 5) portray the long-run impact of public and private expenditures, CO_2_ emissions, energy consumption, and economic growth on the health status of people in the studied region. The public health expenditure positively and significantly affects the health status, which is consistent with the findings of [16–18, 22], but not consistent with the outcomes of [20, 21]. The effect of private health expenditure on health status is also positive and significant, that complies with the results of [20, 22], but does not comply with the findings of [16, 17]. Both the public and private health expenditures increase health status in the studied region due to the broader health care facilities and the extent of effect is greater for private health in the long-run. However,, the CO_2_ emissions significantly reduce the health status (proxied as life expectancy at birth), and this outcome is pertinent to the findings of [28, 29], but not pertinent to [8, 31, 32]. This finding implies that the CO_2_ emissions adversely affect human health by generating environmental pollution and deteriorating air quality. The energy consumption has significant positive impact on human health, which is in the line of the findings of [24–26], but contrary to the results of [56]. This finding implies that the environmentally friendly energy consumption increases the human life by providing the stimulus utilization of modern medical technologies and storing valuable medicines. Similarly, the economic growth (proxied by per capita GDP) also positively and significantly affects the health status, which is relevant to the outcomes of [11, 22, 56]. This outcome denotes that the balanced and sustained economic growth ensures better health facilities and enables people with financial ability for improved medical care in this region. These findings are pragmatic and rational, and have notable empirical significance in the health sector.

Regarding the country-specific short term results in Table 6 (Section 3.6), the negative effect of health care expenditure in various countries may be due to the inefficiency of spending in the health sectors and rampant corruption in health financing. The positive consequence of CO_2_ emissions on the health status of people in some countries may be due to the externality effect of economic development which may generate huge income at the cost of the environment and people may be able to afford better food and housing, but in the long-term, they may affect people’s health negatively. For some countries, the negative coefficients of energy consumption, economic growth and sanitation facilities in the short-term may be due to the immediate adverse impact of these variables by creating pressure on the cost, environment and inefficient utilization of energy and sanitation.

## Conclusion and policy implications

This paper explored the nexus between health status and health expenditures (both public and private), energy consumption and environmental pollution in the SAARC-BIMSTEC region. Using annual data from 2002 to 2017, we have utilized the panel autoregressive distributed lag (ARDL) model, heterogeneous panel causality test, cross sectional dependence test, cointegration test and Pesaran cross sectional dependent (CADF) unit root test to estimate the findings. The acquired results authorize the cointegration among the variables used: public and private health expenditure, energy consumption, and economic growth have positive and statistically significant influence, and environmental pollution has negative and significant effect on the health status of the region in the long-term, but no panel wise significant impact is revealed in the short-term. Bidirectional and unidirectional causality between the studied variables and health status is also revealed. The attained results are theoretically and empirically consistent, and have significant policy implications in the health sector. The policy implication of the findings is: the better and improved health status required to be safeguarded by enunciating the effective policies on both public and private health expenditures, environmental pollution, energy consumption, and economic growth. In this context, the following recommendations should be stressed:

i. *Public-private health expenditure policy mix:* In order to ensure better health care facilities, an adequate, flexible and affordable, corruption free and appropriate health expenditure policy combining public and private initiatives is needed. Establishing a large number of hospitals, supplying a good number of efficient doctors, nurses, and medical equipment and arranging modern health care facilities and better diagnosis instruments – all are essential to guarantee better health.
ii. *Efficient energy policy*: Energy provides the impetus of efficient economic growth which also affects the health status of the people through different channels. By improved living standards, supplying adequate electricity, facilitating manufacturing new drugs, and privileging energy aided medical instruments, energy plays a crucial role in the modern health sector. Therefore, an efficient, affordable, reliable, modern energy policy is urgently required by emphasizing the use of clean and renewable energy in the region.
iii. *Reducing environmental pollution*: Environmental pollution affects human health adversely by generating different viruses and bacteria, which are responsible for various diseases like bronchitis, heart problems, lung problems, and flu related diseases like COVID-19 and many other potentially fatal conditions. In this regard, a green environment policy ensuring by the reduction of CO_2_ emissions should be encouraged to foster longevity.
iv. *Health friendly economic growth policy reforms*: Economic growth and development enables people to earn more and benefit from more modern health facilities; however, the negative external effect of growth activities (increased pollution) may be detrimental to public health. In this context, sustainable and smart health friendly economic growth policy reforms will be helpful to increase people’s life spans throughout the world, and more particularly in the countries that have been mentioned above.

## Data Availability

The datasets used and/or analysed during the current study are available from the corresponding author on reasonable request.

## Footnotes

1 The SAARC-BIMSTEC countries are Afghanistan, Bangladesh, Bhutan, India, Maldives, Nepal, Pakistan, Sri Lanka, Myanmar and Thailand.
